# A Service Held Together in Practice: A Qualitative Study of Interorganizational Collaboration in a Swedish Youth Mental Health and Substance Use Service

**DOI:** 10.1111/inm.70347

**Published:** 2026-09-07

**Authors:** Jofen Kihlström, Martin Salzmann‐Erikson, Irene Hylander, Maria Lindberg

**Affiliations:** ^1^ Faculty of Health and Occupational Studies, Department of Caring Science University of Gävle Gävle Sweden; ^2^ Centre for Research and Development Region Gävleborg/Uppsala University Gävle Sweden

**Keywords:** adolescent health services, delivery of health care, integrated, qualitative research, substance abuse treatment centres

## Abstract

Adolescent substance use frequently co‐occurs with mental‐health difficulties and social adversity. Existing research highlights heterogeneous risk profiles and a paucity of integrated service models that bridge health‐ and social‐care sectors. A qualitative case study was undertaken at the newly established MiniMaria treatment centre in Sweden. Data were gathered through participant observation of 18 steering‐, project‐ and operative‐group meetings, four workplace meetings, and semi‐structured interviews with five staff members. Reflexive thematic analysis was applied iteratively, guided by a conceptual framework of collaborative advantage (power, trust, organizational culture, leadership, goal management). This study adheres to the Standards for Reporting Qualitative Research (SRQR) checklist. Four overarching themes emerged: (1) divergent organizational cultures and power; (2) fragmented goal management; (3) fluctuating trust and leadership; and (4) operational‐level impact on staff. Middle‐management pragmatism compensated for strategic indecision, sustaining service delivery despite resource constraints (for instance absence of a dedicated physician). The MiniMaria project demonstrates that clear, enforceable governance structures are vital for integrated youth services. When senior‐level alignment falters, empowered front‐line managers can preserve continuity of care, but the sustainability of such models hinges on explicit memoranda, shared information systems and ongoing cultural monitoring.

**Abbreviation**
SRQR: standards for reporting qualitative research checklist

## 1 Introduction

Adolescence is a critical period for mental health development, during which social adversity can have lasting effects. Social adversity has been identified as an important contextual factor shaping adolescents' mental health and well‐being (Viner et al. 2012; World Health Organization and Commission on Social Determinants of Health 2008). In addition, previous research has shown that adolescent social adversity is linked to an increased risk of substance use, highlighting its role in influencing youths' mental health outcomes (Wolitzky‐Taylor et al. 2017). Large service‐based cohorts show that substantial proportions of adolescents in treatment report recent substance use. In Ontario, for instance, 22% of youths in community mental‐health care and 37% in residential care report past‐month use, with higher risk among older adolescents and those exposed to self‐harm, abuse, disrupted schooling, or parental substance problems (Aderibigbe et al. 2022). Swedish population data show a social gradient; lifetime cannabis use at ages 17–18 is more common in youths from higher socioeconomic status families, whereas frequent use clusters among lower socioeconomic status youths and co‐occurs with truancy and permissive parental norms (Gripe et al. 2021). Gender differences are small and substance‐specific, with boys reporting slightly higher alcohol quantities and more illicit drug use, while substance‐related problems may peak earlier among girls (Sfendla et al. 2022). Across cohorts, lower parental monitoring, truancy, and minor criminality consistently predict use, alongside co‐occurring psychiatric symptoms, particularly trauma, anxiety, and depression (Dennermalm et al. 2022; Hinckley et al. 2024). Elevated drug use disorder is observed mainly among bisexual girls (Gerdner et al. 2025).

Evidence on treatment interventions to adolescents from the Nordic region is mixed. A narrative review identifies several commonly used interventions for adolescents with substance‐related problems, e.g., the Cannabis Cessation Program, the Icelandic Motivation to Change Inventory for Adolescents, Norwegian Multisystemic Therapy, and Sweden's integrated Maria clinics, many of which are US‐derived or adapted from adult care (Kosonen and Kuusisto 2023). Follow‐up studies show both improvement and persistence of problems (Anderberg et al. 2021; Dahlberg et al. 2022). Gender differences are evident at intake and years after treatment, with distinct risk and prognostic factors for girls and boys (Dahlberg et al. 2022). Staff reports from Swedish specialized services, including Maria clinics, indicate high competence but also role‐specific training needs, underscoring the importance of coordinated, context‐sensitive service development (Kapetanovic et al. 2025).

Integrated youth services, such as Maria clinics, are promoted to address fragmented care pathways. A narrative synthesis of youth mental health models identifies common components mapped to the World Health Organizations health system building blocks and proposes six levels of integration, from coordination to fully integrated practice. Key elements include multidisciplinary teams, care coordination, shared information systems, digital and financing arrangements, leadership, governance, and shared values (Hodgins et al. 2024). Empirical evidence from a randomized trial shows that integrated collaborative care achieved outcomes comparable to hospital outpatient care while improving access and reducing psychiatrist visits (Henderson et al. 2025).

In Sweden, statutory coordinated individual plans aim to bridge health and social care, yet reviews show low to moderate integration, uneven mandates, limited shared problem formulations and long‐term goals, and weak user involvement despite co‐location and regular meetings (Matscheck and Piuva 2022). MiniMaria clinics are a concept where municipalities and county councils work together in integrated treatment settings with the aim of bridging the gap between healthcare and social services. The overarching idea is to work integrated instead of offering their services in a co‐located setting (Kvarmans et al. 2026). Studies of MiniMaria settings show how frontline practice is shaped by scientific, structural, professional, and procedural legitimizing logics framing interventions and cannabis as a policy problem (Ekendahl et al. 2018). Young people's narratives reveal tensions between responsibility, identity work, and problem‐burdened accounts linked to resistance or compliance in treatment (Ekendahl and Karlsson 2022). These dynamics help explain why integration may stall when mandates, legitimizing logics, and routines are misaligned (Ekendahl et al. 2018). Evidence from the planning phase of a Swedish MiniMaria clinic similarly indicates that progress depends on ongoing collaborative processes rather than formal structures (Lindberg et al. 2024). Building on this, the present study examines how integration is enacted once a MiniMaria clinic becomes operational, analysing the interorganizational collaboration underlying its establishment through a theoretical collaboration framework.

## 2 Theoretical Framework

Drawing on Huxham and Vangen's theory of Collaborative Advantage, this study applies an analytical framework linking empirical observations to key concepts in inter‐organizational collaboration. The framework examines how collaborative advantage and collaborative inertia emerge through processes related to power, trust, organizational culture, leadership, and goal management (Huxham and Vangen 2013). Gray (1985) defines collaboration as stakeholders moving beyond differences to develop mutually beneficial solutions. Yet around half of collaborative initiatives fail during implementation due to misaligned incentives and self‐interest (Lunnan and Haugland 2008; Prashant and Harbir 2009). Cruz (2023) frames collaboration as defining problems, identifying solutions, and implementing agreed resolutions, though inter‐organizational efforts often experience inertia and frustration (Vangen and Huxham 2005).

The collaborative Advantage framework analyses collaboration through five interrelated elements: power, organizational culture, trust, leadership, and goal management. Power involves mobilization of resources within unequal relationships, creating negotiation and uncertainty as actors pursue their own goals (Cruz 2023). Organizational culture may stimulate creativity but can also generate conflict, while trust develops dynamically through expected benefits and an initial willingness to take risks (Huxham and Vangen 2013). Effective leadership fosters goal clarity, participation, innovation, and interprofessional coordination (Lyubovnikova et al. 2015; McCallin 2003; Mickan and Rodger 2000; Thylefors et al. 2005). Leadership also plays a central role in balancing power, aligning stakeholder agendas, and coordinating contributions across organizations (Huxham and Vangen 2013). Goal management involves identifying, negotiating, and aligning objectives while monitoring progress to sustain collaboration and prevent inertia. Central to this process is goal alignment, through which stakeholders coordinate efforts toward shared objectives (Gulati et al. 2012; Huxham and Vangen 2013).

## 3 Aim

This article primarily aims to explore how the inter‐organizational collaboration influenced both the establishment of the MiniMaria treatment centre and the unfolding of its service delivery in practice. The guiding research question was ‘How did collaborative processes between the two stakeholders shape the establishment and delivery of services at the treatment centre?’

## 4 Methods

This study is part of a broader research initiative examining collaboration between a municipality and a county council in the care of youths with substance use problems, such as alcohol, drugs or gambling for money, and their families. It explores interorganizational collaboration and quality improvement to promote person‐centred care, incorporating the experiences of youths and families to provide a holistic view of MiniMaria's services. The objective was to inform policy and practice development at county and national levels. Throughout the project, the four researchers observed meetings held by the steering, project, and operative groups between 2023 and 2025, while two authors also conducted interviews. The steering group was responsible for strategic decisions, the project group for operational decisions, and the operative group for practical issues related to establishing and running the treatment centre. The steering group approved a project plan formalizing collaboration through a memorandum of understanding. As described in a previous article (Lindberg et al. 2024), the agreement followed extensive negotiations regarding responsibilities and staffing, which influenced the opening of the centre. The study was reported in accordance with the Standards for Reporting Qualitative Research (SRQR) checklist (O'Brien et al. 2014). Protons LLM Lumo assisted with language editing under author supervision.

### 4.1 Design and Setting

An exploratory design, incorporating a participatory research approach focused on a joint process of knowledge production to generate new insights for both researchers and participants, was adopted for this qualitative study. The municipality and county council were selected through purposive sampling, based on the proximity and accessibility of the field. The project leader, responsible for establishing the MiniMaria treatment centre, initiated contact with the university requesting collaboration to follow the processes. The project leader both supported field access and contributed as a participant.

### 4.2 Participants

Before the researchers entered the field, two primary groups had been established, a steering group and a project group. The steering group consisted of six administrators in senior positions, representing both the county council and the municipality. The project group comprised 13 individuals, primarily in first‐line and middle management positions from both organizations. Additionally, a third group, the operative group, was formed, consisting of seven representatives in managerial roles or team leaders. All staff at the newly opened MiniMaria treatment centre participated. As all eligible participants and relevant meetings during the study period were included, additional sampling was considered unlikely to provide substantial new information.

### 4.3 Data Collection

Before data collection began, all included informants received oral and written information about the research project and they all gave their consent to participate in the study. For this specific article, we attended six meetings with the steering group (2023–2025), five meetings with the project group (2023–2025), and seven meetings with the operative group (2024–2025). At all meetings, the researchers took extensive notes based on the conversation content, and one meeting was also audio recorded. After each meeting, the researchers convened to discuss the meeting: what were our overall impressions, what topics were discussed, and what decisions were made? How did participants address one another, and what processes were evident? We wrote analytical field notes based on our joint discussions of what we observed and our interpretations of the conversations. Observations were also conducted by the first author during four workplace meetings involving the two managers and the five staff at the MiniMaria treatment centre. During these observations, notes were taken primarily on organizational issues related to the opening of the centre. Furthermore, the first author conducted three interviews with staff members (*n* = 5), including two paired interviews and one individual interview, to explore whether staff perceived sufficient conditions and resources to support youths attending the treatment centre. Interview topics included perceptions of the implementation process, division of labour, leadership, awareness of the project plan preceding the centre's opening, and views on governance by the principal stakeholders. The interviews were semi‐structured and exploratory in nature; they were audio recorded and transcribed verbatim to support subsequent analysis. Both the transcriptions and audio recordings were stored on the university's server to ensure all researchers had access to the data. To ensure confidentiality, participants were de‐identified using numerical codes.

### 4.4 Data Analysis

Data collection and analysis were conducted concurrently, resulting in a recursive and non‐linear process. The interview transcripts and the analytical field notes were read repeatedly and analysed using reflexive thematic analysis (Braun and Clarke 2019, 2021). The analytic process began with immersive familiarization, followed by systematic, organic coding. Codes were treated as interpretative tools rather than descriptive labels, capturing both semantic and latent meanings. These were iteratively reviewed and grouped into clusters of shared meaning. Themes were developed through interpretative engagement with these clusters and conceptualized as patterns of shared meaning underpinned by a central organizing concept (Clarke and Braun 2017). Identified themes were integrated with existing theories and concepts, thereby enhancing the theoretical depth and robustness of the analysis (Timmermans and Tavory 2012; van Hulst and Visser 2025; Vila‐Henninger et al. 2024). The researchers reflected continuously on their assumptions and potential influence throughout the research process, and no substantial influence on the findings was considered evident.

### 4.5 Rigour

To ensure methodological rigour, several strategies were employed in line with established qualitative criteria (Braun and Clarke 2021; Yardley 2015). Reflexivity was embedded throughout the process. Prolonged engagement in the field enabled the research team to develop contextual understanding, while rich descriptions and illustrative data extracts supported transferability. Dependability and confirmability were addressed through collaborative analysis. All researchers read the transcripts and participated in iterative discussions of field notes and coding decisions. Although one researcher conducted staff interviews and attended workplace meetings, the rotation of researchers during other data collection activities ensured diversity of perspectives and analytic consistency over time.

## 5 Results

Based on the analysis, four themes were identified: (1) Divergent organizational culture and power, contrasting county council and municipality structures; (2) Fragmented goal management, reflecting stalled implementation and limited interfaces; (3) Fluctuating trust and leadership, revealing eroding inter‐organizational trust and solution‐oriented project leadership; (4) Operational‐level impact on staff, including start‐up challenges, missing physician capacity, and technical constraints. The themes are presented below and illustrated with excerpts from the empirical material.

### 5.1 Divergent Organizational Culture and Power

Observations showed that the county council had limited capacity to enforce decisions across relatively autonomous branches, unlike the smaller, more integrated municipality. Distinct organizational cultures were evident in interviews and meetings. Resistance from middle management, questioning MiniMaria's relevance to core missions and allocated resources, constituted the main obstruction, generating conflict within the steering group. For example, a conflict emerged when the primary care representative stated that care for individuals with drug and alcohol addiction was outside their remit. The project group demonstrated a solution‐oriented culture focused on practical problem‐solving and collaboration, without challenging the memorandum. The operative group similarly emphasized problem‐solving and information‐sharing, fostering effective case handling. In contrast, staff at the MiniMaria treatment centre were in an early start‐up phase without a shared organizational culture. Efforts were still underway to define day‐to‐day operations. Ongoing conflict and the steering group's limited authority contributed to staff uncertainty regarding commitment from stakeholders involved. In the words of one participant:

I find that so strange, that this bug still persists when we talk about levels of care. But in the project plan it clearly states Early Detection, Abuse and Dependency – I mean, it's not preventive work if someone has developed a dependency. That's a treatment intervention. But it's as if they haven't read the project plan. I mean, they're the ones who commissioned it, and then they say something else, and that's something we've struggled with a bit (Interview staff member; 2025)

The asymmetrical power dynamics between the two stakeholders became evident during meetings where conflicts between the county council and the municipality remained unresolved. Despite the provisions outlined in the memorandum of understanding, the municipality were unable to hold the county council accountable for its commitments. The organizational structure and relative authority enabled the county council to deviate from previously agreed‐upon obligations.

### 5.2 Fragmented Goal Management

Failure to adhere to the memorandum's goals forced a return to negotiation, undermining goal management. Conflict arose over MiniMaria's status as pilot or permanent service, with middle managers viewing it as misaligned with established organizational routines and structures. During a meeting of the steering group, it was noted:

Some middle managers argued that MiniMaria should focus exclusively on prevention, and that cases involving established substance abuse and mental health issues should be referred directly to specialist care, despite the memorandum stating that MiniMaria was to work with both preventive efforts and individuals with developed dependency. (Field note; 2024)

The project group raised the physician issue in the steering group, but it remained unresolved, resulting in no appointment and requiring first‐line managers to devise alternative solutions in line with the project plan. Apparent steering group consensus concealed conflicts driven by divergent middle‐management interests, as illustrated in field notes:

Regarding the appointment of a physician for four hours per month, a middle manager contrasted two patient groups and argued against allocating medical resources due to difficulties in recruiting physicians, claiming that another patient group was in greater need of available physicians. (Field note; 2024)

Steering group conflict and indecision shifted responsibility to first‐line managers, while the operative group maintained a practical focus. Strategic discussions about appointing a physician were avoided, shielding daily work from unresolved issues. Prolonged indecision hindered adherence to the project plan and generated staff frustration, raising questions about the steering group's commitment, as one participant expressed:

Sometimes you get a bit worried and think, what the hell, what if they pull out? What if they decide something else, what if a directive comes that… I mean, you don't really feel sure about what they do next… (Interview staff member; 2025).

Staff adopted a solution‐oriented approach, while conflicting agendas in the steering group impeded goal management. A lack of reciprocity fostered perceptions of county council ambivalence and reduced credibility at lower organizational levels.

The challenges encountered and managed by the project group effectively shielded the operative group from having to address these issues. By absorbing and resolving strategic and organizational tensions, the project group enabled the operative level to maintain its focus on practical collaboration and service delivery. The group most directly challenged by how the project's goals were formulated at the operational level was the staff.

I would really wish that, at all levels, people would think now we have the chance, we have three years to actually step outside our boundaries. This is a pilot project, and the point of those is unless I'm completely stupid that you get to try new ways of working and then draw conclusions from that. Was this good or not? Should we change something? Then we can't be so terribly afraid to take a leap and try something new. (Interview staff member; 2025)

As viewed in the quote staff consistently reported that the resources and routines guiding their work did not align with the objectives outlined in the project plan. During interviews, staff frequently expressed doubt as to whether middle managers within the steering group had read or understood the project plan or even recognized that the initiative was intended as a pilot project.

### 5.3 Fluctuating Trust and Leadership

Trust and leadership were inseparable, shaping decisions and daily operations. The steering group's inability to enforce agreements created frustration, undermining confidence among stakeholders and weakening the leadership it was expected to provide.

One staff member expressed concern that the county council and municipality expected to the new service to receive as many visitors as established services. Given limited trust in higher management's understanding of start‐up challenges, these expectations were perceived as potentially threatening to the initiative. (Unrecorded interview staff member; 2025)

The main issues undermining trust and leadership were the allocation of a physician and the role of primary care, affecting multiple levels and perspectives. While the municipality consistently implemented steering group decisions, they expressed frustration with the county council's inability to follow through. Paradoxically, these leadership shortcomings and lack of mutual trust strengthened cohesion within the project group, as members focused on fulfilling the project plan. Collaborative problem‐solving and constructive dialogue fostered trust, enhanced leadership capacity, and motivated staff. Operational staff observed that managerial collaboration produced tangible results, creating shared understanding of routines and case management to best serve visitors. At one project group meeting it was noted:

The managers showed a strong commitment to resolving how MiniMaria could function as a hub for visitors supporting them in accessing specialist care while maintaining ongoing contact throughout the process. (Field note; 2024).

This visible cooperation among managers contributed to a sense of coherence and strengthened the conditions for effective service delivery. Staff, aware of higher‐level leadership limitations, valued their immediate managers' efforts, reporting trust in their leadership, confidence in performing high‐quality care, and that visitors were supported professionally and respectfully.

From my perspective, I really feel that one of the managers is deeply committed to this initiative. She's passionate about the work and has fought for a long time to make MiniMaria a reality. And if there's something she believes needs to happen, she won't let it go. She genuinely believes in this, and I really feel that we benefit from that. (Interview staff member; 2025)

### 5.4 Operational‐Level Impact on Staff

The initial phase of the MiniMaria treatment centre faced several challenges, including early staff turnover and delays in recruiting a nurse, which left only one position filled for an extended period. Despite these setbacks, staff collaborated closely across multiple county council departments, fostering a sense of capability and purpose in supporting visitors. Structural limitations, such as the lack of a shared medical records system and calendar access, required frequent briefings, which were time‐consuming but enhanced team cohesion. Staff were granted autonomy to manage ambiguities in the project plan, enabling practical solutions and reinforcing collaboration both within and across organizational boundaries. These conditions allowed staff to deliver high‐quality care, with visitor appreciation serving as a motivating factor for continuous service development, even in the face of ongoing frustrations. Two staff members reflected on visitors' experiences at MiniMaria:

I think we have visitors who come for their appointments and want to recommend us to their friends. Yes, oh absolutely, and to their family members too …//… So I don't think they even consider that there are different responsible authorities, I don't think they reflect on that. (Interview staff member; 2025)

## 6 Discussion

This study explored how inter‐organizational collaboration shaped both the establishment of the MiniMaria treatment centre and the delivery of its services. Processes related to power affected decision‐making and the ability to implement agreements; organizational culture shaped how teams coordinated their work; leadership balanced strategic direction with everyday facilitation; trust grew through practical collaboration but was weaker at higher levels; and goal management required continuous negotiation to align objectives across organizations. At the operational level, however, staff demonstrated strong commitment and close collaboration, enabling the treatment centre to function despite ongoing challenges. Taken together, these factors help explain how collaboration may support practical service delivery while also creating challenges at the strategic level, and why service continuity could be preserved even when integration remained fragile.

The findings suggest that power was unevenly distributed, with limited authority at the steering‐group level and high autonomy in operational units. This may have constrained the translation of formal agreements into action, contributing to collaborative inertia rather than advantage (Huxham and Vangen 2013). While autonomy enabled operational problem solving, weak enforcement capacity limited strategic coordination, which may partly explain why issues were repeatedly renegotiated rather than resolved. Research shows that power asymmetries can undermine collective decision‐making, and collaborative governance theory emphasizes the need for authority to convert consensus into joint action (Emerson et al. 2011). Cruz (2023) highlights that collaborative settings are characterized by unequal power relations, where uneven resources fuel negotiation, tension, and uncertainty as actors pursue their own goals. These findings align with research showing that collaborative initiatives often fail because of misaligned incentives and self‐interested behaviour (Lunnan and Haugland 2008; Prashant and Harbir 2009). At MiniMaria, repeated goal renegotiations and limited steering‐group authority illustrate these challenges. Rather than reflecting poor communication alone, this pattern may suggest that the collaboration lacked sufficient authority to convert agreement into action. To improve collaboration, organizations may benefit from clarifying decision‐making roles and creating mechanisms for enforcing agreements across units.

Organizational culture varied across collaborative levels, shaping how integration was enacted in practice. Solution‐oriented norms developed in the project and operative groups, facilitating everyday coordination, while the treatment centre remained in an early phase of cultural formation. From a Collaborative Advantage perspective, cultural alignment emerges through repeated interaction rather than formal agreements (Huxham and Vangen 2013). Research shows that shared norms can facilitate collaboration within groups while reinforcing fragmentation across organizational boundaries (Schein 2010), and studies of health and care integration initiatives similarly highlight an overreliance on structural approaches to change, with insufficient attention to relational factors such as leadership and interpersonal collaboration needed for sustained transformation of services and inter‐organizational practices (Olaitan 2025). This resonates with the present findings, where collaboration within operational teams functioned well, while challenges at higher organizational levels influenced coordination and decision‐making across the two stakeholders. One possible interpretation is that cultural alignment developed through shared work at the operational level, while remaining weaker where collaboration relied more heavily on formal mandate than on repeated interaction. Early efforts to build shared practices and routines can help reduce fragmentation and support more effective collaboration.

Leadership was constrained by unclear mandates and limited enforcement capacity, undermining confidence in strategic governance while shifting coordination responsibilities to operational levels. From a Collaborative Advantage perspective, leadership was enacted more through facilitation and conflict management than through strategic direction (Huxham and Vangen 2013). Research on collective and distributed leadership suggests that leadership emerges through interaction rather than formal roles, although unclear mandates may still hinder coordination despite effective operational leadership (Fournier et al. 2022). Previous research further shows that clear leadership supports coordination, engagement, innovation, empowerment, and shared decision‐making in interprofessional teams (Lyubovnikova et al. 2015; McCallin 2003; Mickan and Rodger 2000; Thylefors et al. 2005). The present findings therefore suggest not an absence of leadership, but a mismatch between strategic leadership capacity and operational leadership practice. Clarifying leadership roles and responsibilities may strengthen confidence and support more consistent decision‐making across organizational levels.

Trust developed unevenly, weakened by unresolved strategic conflicts but strengthened through collaborative problem‐solving at operational levels. In line with Collaborative Advantage theory, trust appears to emerge through repeated interaction rather than formal agreements (Huxham and Vangen 2013). This suggests that trust was not a single feature of the collaboration, but something produced differently across organizational levels. Thus, structured opportunities for communication and joint problem‐solving can help strengthen trust across all levels of collaboration.

Goal management was challenged by repeated renegotiations of objectives, as misalignment between the project plan and organizational routines undermined sustained alignment. During data collection, the steering group met the project group on only two occasions, which may have contributed to limited attention to decisions perceived as critical by the project group. This likely reduced the opportunities for operational concerns to be represented and addressed. This became evident when the project group had to manage the consequences of delegating responsibility for a dedicated physician to primary care services. From a Collaborative Advantage perspective, this suggests weak goal management, where formally agreed goals lacked anchoring across organizations (Huxham and Vangen 2013). The project group also developed its own procedures for determining when visitors required specialist care, since standard protocols mandated such care in cases involving psychoactive substance dependency, a need the MiniMaria treatment centre was intended to mitigate. Nevertheless, staff shifted focus from resource constraints to delivering care outlined in the project plan. Research emphasizes that goal alignment is a continuous process of coordinating actions toward shared objectives (Gulati et al. 2012). As Cruz (2023) notes, collaborative settings are marked by uncertainty as actors pursue their own goals, highlighting the importance of deliberate alignment and monitoring. Taken together, this suggests that goal management was not a settled achievement but an ongoing accomplishment requiring more active maintenance than the steering structure provided. Embedding shared goals into daily routines and ensuring all partners understand their roles may reduce misalignment and support collaborative momentum.

Previous research indicates that successful implementation of improvement initiatives depends on structured methodologies, leadership engagement, skilled facilitation, and effective teamwork (Hibbert et al. 2021; Varatharasan et al. 2024). Although key elements consistent with Hodgins et al. (2024), such as multidisciplinary teams, care coordination across care levels, and leadership expressing shared values, were in place, the integrated service could have been implemented with greater consistency between stakeholders. This suggests that integrative components alone were insufficient; equally important was how consistently they were supported across organizational levels. Using an analytical framework such as the Youth Integration Project Framework (Hodgins et al. 2024) might have facilitated coordination, alignment, and collaboration during MiniMaria's establishment. Integrated care, including co‐located mental health and alcohol and other drug services and collaborative care models, has been associated with improved treatment engagement and outcomes for youth with co‐occurring substance use and mental health problems (Glover‐Wright et al. 2023; Henderson et al. 2025). From the youth perspective, effective services require availability across the care continuum, holistic responses, and respectful, empathetic delivery (Marchand et al. 2022). These findings highlight that accessibility and respectful care remain central even when organizational arrangements are unsettled. This underscores the need for tailored training aligned with professionals' educational backgrounds and organizational contexts to strengthen interprofessional collaboration and treatment outcomes (Kapetanovic et al. 2025; Searby et al. 2025). Service integration also depends on adequate resources and interdisciplinary approaches, while being constrained by staff attitudes, low mental health literacy, and organizational barriers such as territorialism (Searby et al. 2025).

## 7 Limitations

The study has limitations regarding transferability, as the participants consist of a limited cohort of individuals involved in a single project, thereby excluding outside perspectives. Although the interview sample was small, its adequacy was supported by the participants' specific, first‐hand knowledge of the establishment process and by the integration of interview data with longitudinal observations across several organizational forums, which strengthened the information power of the material (Malterud et al. 2016). This may constrain the applicability of the findings to similar projects. The interpretative analysis is dependent on the researchers' understanding and selected theoretical lenses. While none of the authors have organizational theory as their primary research field, one researcher had concurrent employment with one of the stakeholders, which provided valuable pre‐understanding of the field but also necessitated heightened reflexivity regarding potential biases. In line with this, we chose not to fully specify interview dates or individual roles in the data excerpts to ensure strict confidentiality. Additionally, one interview was not recorded due to a technical error, though extensive notes were taken immediately following the interview. Despite these limitations, the study provides nuanced insights into how the interplay between two stakeholders functions.

## 8 Conclusion

Collaboration did not unfold as a stable or linear implementation process. Formal agreement at senior level enabled the launch but did not secure sustained integration once the service became operational. Instead, the findings point to persistent tension between strategic intent and everyday practice, evident in the unresolved physician issue, unclear mandates, and slippage in goal alignment. A key contribution is showing why implementation may appear successful at the point of care while remaining structurally fragile: trust and leadership weakened at the strategic level, yet middle managers and frontline staff generated continuity, flexibility, and shared purpose to keep the service functioning. The service relied on compensatory labour and negotiation rather than stable organizational support. Although this is a single case, the study suggests that integrated youth services are unlikely to be sustained by co‐location or formal partnership alone. Durable integration appears to require enforceable governance, shared information systems, and routine cross‐level accountability.

## 9 Relevance for Clinical Practice

In everyday clinical work, the question is seldom whether a youth service is formally integrated, but whether young people and their families experience it as one. The findings suggest this depends on easily neglected conditions, such as clear responsibilities, agreed referral routes, access to medical input, and time for professionals to discuss cases. When these conditions are unclear, staff may still maintain care through informal fixes, personal commitment, and negotiation. Although this may protect young people from organizational gaps in the short term, it also makes services vulnerable. Models relying heavily on staff goodwill risk becoming uneven when staff leave, workloads rise, or decisions remain unresolved. Integrated youth services therefore need more than enthusiastic teams. First‐line managers and staff need authority to act and reliable routes for escalating problems that cannot be solved locally. Shared routines, documentation practices, and regular interprofessional meetings should be treated as part of care itself, not background administration.

## Author Contributions

Jofen Kihlström, Martin Salzmann‐Erikson, Irene Hylander, and Maria Lindberg created the study design, framing, and conceptualization and formulated the aim of the study. Jofen Kihlström, Martin Salzmann‐Erikson, Irene Hylander, and Maria Lindberg were all involved in data collection through observations, while Jofen Kihlström collected the interview data. Jofen Kihlström and Martin Salzmann‐Erikson drafted the background. Jofen Kihlström and Martin Salzmann‐Erikson jointly analysed, coded, and interpreted the data, and Jofen Kihlström, Martin Salzmann‐Erikson, and Maria Lindberg drafted the methods and results sections in collaboration with Irene Hylander. Maria Lindberg drafted the discussion. Jofen Kihlström, Martin Salzmann‐Erikson, Irene Hylander, and Maria Lindberg jointly developed the manuscript throughout the writing process. All authors read and approved the final manuscript.

## Funding

This work was supported by the University of Gävle, Sweden and Region Gävleborg, Sweden. The funder had no role in the design of the study, in the collection, analysis, or interpretation of data, in the process of writing and publishing the manuscript. Open access funding was provided by the University of Gävle.

## Disclosure

The first author was employed by the included county council as an analyst in community medicine, but his ordinary role was organisationally and functionally separate from MiniMaria. He was not employed in the Research & Development Welfare unit associated with the project leader, had no operational role in the service, and field access was arranged through the project leader's collaboration with the university rather than through his employment. The research team nevertheless discussed whether his institutional affiliation could influence interpretation. On one occasion, when he was addressed in his analyst role during a meeting where findings were reported, he explicitly clarified that he was present as a researcher and responded solely with reference to the study findings.

## Ethics Statement

The study protocol was approved by the Swedish Ethical Review Authority (Dnr 2023–03479‐01), and informed written consent was obtained from all participants involved in the study.

## Consent

The authors have nothing to report.

## Conflicts of Interest

The authors declare no conflicts of interest.

## Data Availability

The datasets generated and analysed during the current study are not publicly available due to privacy or ethical restrictions but are available from the corresponding author on reasonable request. A data management plan was registered in DMPonline (reg. no 123662).
